# Establishment and research progress of animal models for intervertebral disc degeneration

**DOI:** 10.1515/biol-2025-1295

**Published:** 2026-03-02

**Authors:** Cong Zhang, Rui Sun, Qing Jiang

**Affiliations:** Division of Sports Medicine and Adult Reconstructive Surgery, Department of Orthopedic Surgery, Nanjing Drum Tower Hospital, Affiliated Hospital of Medical School, Nanjing University, 210008 Nanjing, Jiangsu, China; Department of Spine Surgery, Zhongda Hospital, School of Medicine, Southeast University, 210008 Nanjing, Jiangsu, China

**Keywords:** intervertebral disc degeneration, low back pain, nucleus pulposus, annulus fibrosus, animal model

## Abstract

Low back pain associated with intervertebral disc degeneration (IDD) is a prevalent condition in clinical practice, significantly impacting patients’ work and quality of life. Animal models are indispensable for IDD research, offering crucial tools to investigate the molecular mechanisms of disease onset and progression, as well as to evaluate potential therapeutic interventions. Current animal models for IDD include intervertebral disc injury, spontaneous degeneration, mechanically induced, and chemically induced models, each exhibiting unique strengths and limitations in mimicking the pathological features of human IDD. Despite these advancements, existing models continue to struggle with replicating the long-term, progressive nature of degeneration and the heterogeneity observed in human patients. With the emergence of bioengineering techniques and molecular imaging, novel approaches to model construction and evaluation have opened new avenues for IDD research. This review systematically synthesizes current strategies for constructing IDD animal models, their application characteristics, and associated challenges, while also projecting future research directions. The aim is to provide guidance for optimizing model selection and accelerating translational research in the field of IDD.

## 1 Introduction

IDD is a common degenerative disorder of the musculoskeletal system and a major cause of chronic low back pain, severely affecting the quality of life of patients and imposing a significant economic burden on families and society [1], 2]. About 20 % of adolescents have mild IDD, and 80 % have experienced back pain during their lifetime [3]. Currently, the pathogenesis of IDD remains incompletely understood, resulting in a lack of standardized, effective treatments for IDD-related diseases. Due to the limited availability of human experimental materials, establishing reliable animal models of IDD is crucial. These models offer a vital approach for elucidating the underlying mechanisms of disc degeneration and serve as valuable platforms for evaluating potential therapeutic interventions [4], [5], [6].

This review provides a comprehensive overview of currently employed animal models for IDD, comparing their respective advantages, disadvantages, applicability, and limitations. We recommend that researchers carefully select the most appropriate animal model for their IDD studies, based on factors such as the experimental species, the desired experimental duration, and the specific pathological features being investigated. Future research should focus on refining model construction standards, incorporating multi-omics technologies to elucidate the intricate mechanisms of IDD, and developing more clinically relevant evaluation systems for emerging intervertebral disc regeneration therapies.

## 2 *In vitro* model

### 2.1 Intervertebral disc cell model

The intervertebral disc cells were cultured *in vitro* by cell biology technology, and the characteristics of their growth, proliferation and differentiation were observed. The model was treated with external conditions to explore the effect of single factor on IDD. Wang et al. used lipopolysaccharide and adenosine triphosphate to induce nucleus pulposus cells (NPCs) degeneration model *in vitro* [7]. Herrera et al. used monolayer and three-dimensional culture to study human intervertebral disc cells, which showed a significant increase in type I and type II collagen content in three-dimensional culture. Compared to monolayer culture, three-dimensional culture provides a more complex culture environment and can reduce the loss of cell viability and unstable protein expression [8]. The co-culture of NPCs with BMSCs in a three-dimensional environment can increase inflammatory resistance and autoimmune regulation [9].

However, neither monolayer nor three-dimensional *in vitro* culture fully replicates the complex human environment, potentially overlooking the influence of mechanical and other crucial factors on IDD [10], 11]. The absence of a functional extracellular matrix in *in vitro* settings hinders cell proliferation, compromises the maintenance of a stable cellular phenotype, and can even lead to dedifferentiation or a complete loss of original cellular function [12], 13].

### 2.2 Intervertebral disc tissue model

*In vitro* models of intervertebral disc (IVD) tissue can be broadly categorized into those that incorporate the cartilage endplate (CEP) and those that do not. The IVD comprises the nucleus pulposus (NP), annulus fibrosus (AF), and CEP. The CEP serves as a crucial pathway for IVD nutrition and metabolism, maintaining IVD integrity and limiting tissue expansion. Preserving the CEP in *in vitro* models can limit tissue swelling and ensure adequate nutrient supply, thereby better mimicking the *in vivo* environment. For instance, researchers have successfully infected rat IVD tissue *in vitro* with an adenovirus vector carrying green fluorescent protein (GFP), observing GFP expression in both the CEP and outer AF for at least 14 days. This underscores the potential of using adenoviruses as vectors for gene therapy in IVD repair and slowing disc degeneration, offering a more precise approach for managing and treating degenerative disc disease [14]. A novel ex vivo goat IDD model involves pre-loading under simulated physiological load conditions during the day, followed by enzymatic degradation of lumbar goat intervertebral disc through injection of collagenase and chondroitinase ABC. After digestion, simulated physiological loading was performed on intervertebral disc for 7 days, and it was found that the extracellular matrix components decreased, while interleukin-1 *β*, −8, and VEGF continued to increase. The observed changes are consistent with the changes in human IDD [15].

The comprehensive tissue culture model offers the advantage of utilizing the inherent structure of the IVD, allowing cells to reside within their natural matrix and preserving the morphological integrity of the tissue, including cell-cell interactions [16]. Functioning as a bridge between *in vivo* and cellular *in vitro* models, the IVD tissue model provides a valuable experimental platform. This allows researchers to study the tissue’s response to external stimuli and observe the histological changes occurring in both healthy and degenerated discs [4], 17], 18].

## 3 *In vivo* models

### 3.1 Intervertebral disc injury model

This model predominantly induces IDD by surgically damaging the NP, AF, and CEP. The fine needle puncture method is a common choice, favored for its ease of use, consistent results, and the ability to finely control the extent of the induced damage.

#### 3.1.1 Annulus fibrosus and nucleus pulposus injury

Glaeser et al. showed that anterior disc injury using an 18G needle induced severe IDD and mechanical hypersensitivity, whereas the 21G needle resulted in moderate degeneration without increased pain sensitivity [19]. In a separate approach, a 2 mm channel was drilled through the CEP into the NP to perform a partial nucleus pulpectomy, creating an animal model of progressive IDD while maintaining an intact AF. This model is beneficial for investigating NP regeneration and repair, as well as early-stage, mild IDD [20]. In our previous experiments, we targeted the rat coccygeal 5–6, 6–7, 7–8, and 8–9 discs. These were percutaneously punctured using a 21 G needle with a stopper set to a depth of 5 mm; the needle was then rotated 360° and held for 30 s. Four weeks post-operatively, we observed significant loss of disc height, accompanied by an increase in Pfirrmann grades [21], 22].

**Figure 1:**
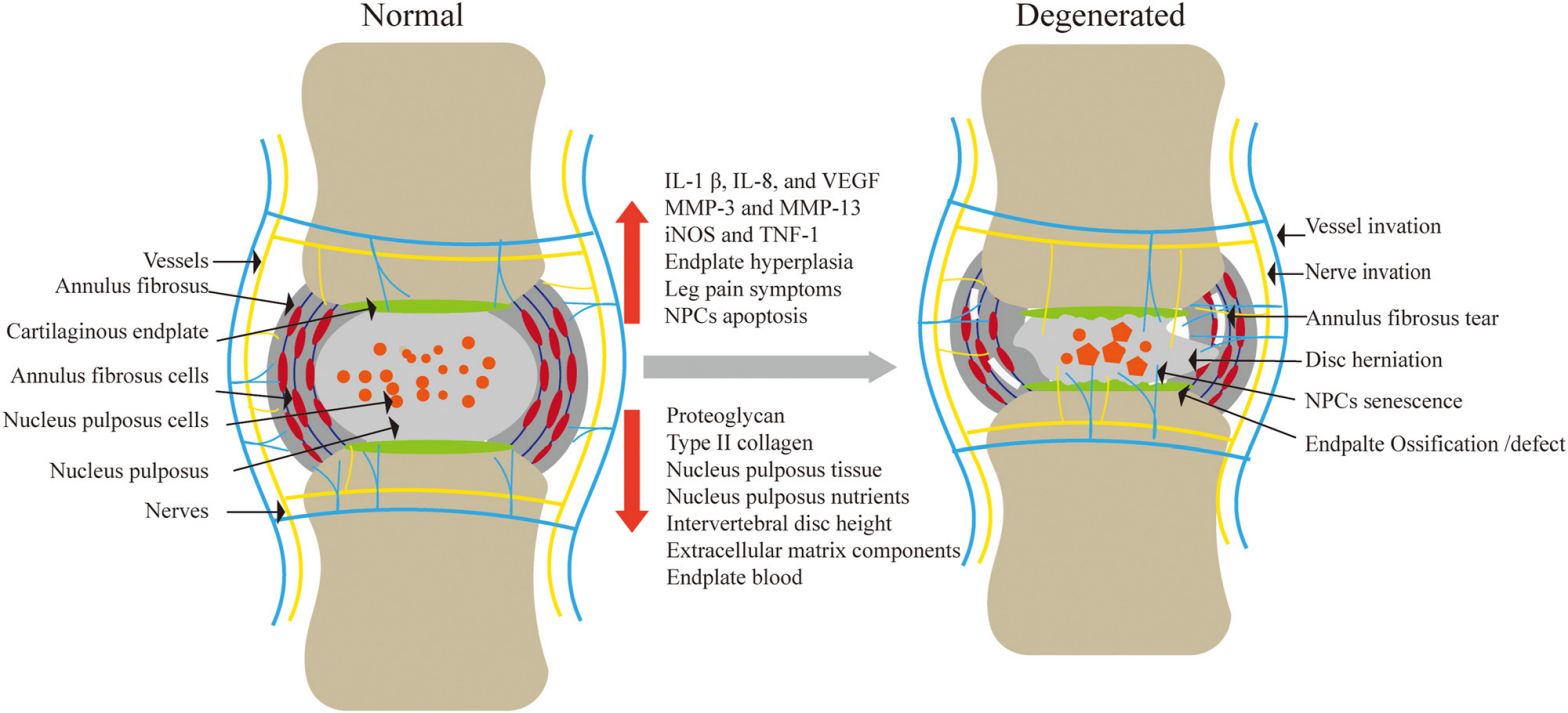
Degenerated intervertebral discs show the ingrowth of blood vessels and nerves, aging of nucleus pulposus cells, tearing of the annulus fibrosus, and increased levels of inflammatory factors.

In addition, as the structural integrity of the disc is disrupted, nerves and blood vessels can enter the NP through the torn AF [23], 24]. This nerves and blood vessels invasion is associated with low back pain (Figure 1). It was found that after AF puncture in mice, tears in the AF occurred, with loss of NP tissue and an increase in radiating leg pain symptoms. Increased nerve distribution and painful nerve fibres density in degenerating discs were also found, suggesting that the process of disc degeneration is accompanied by nerves and blood vessels invasion [25].

**Figure 2:**
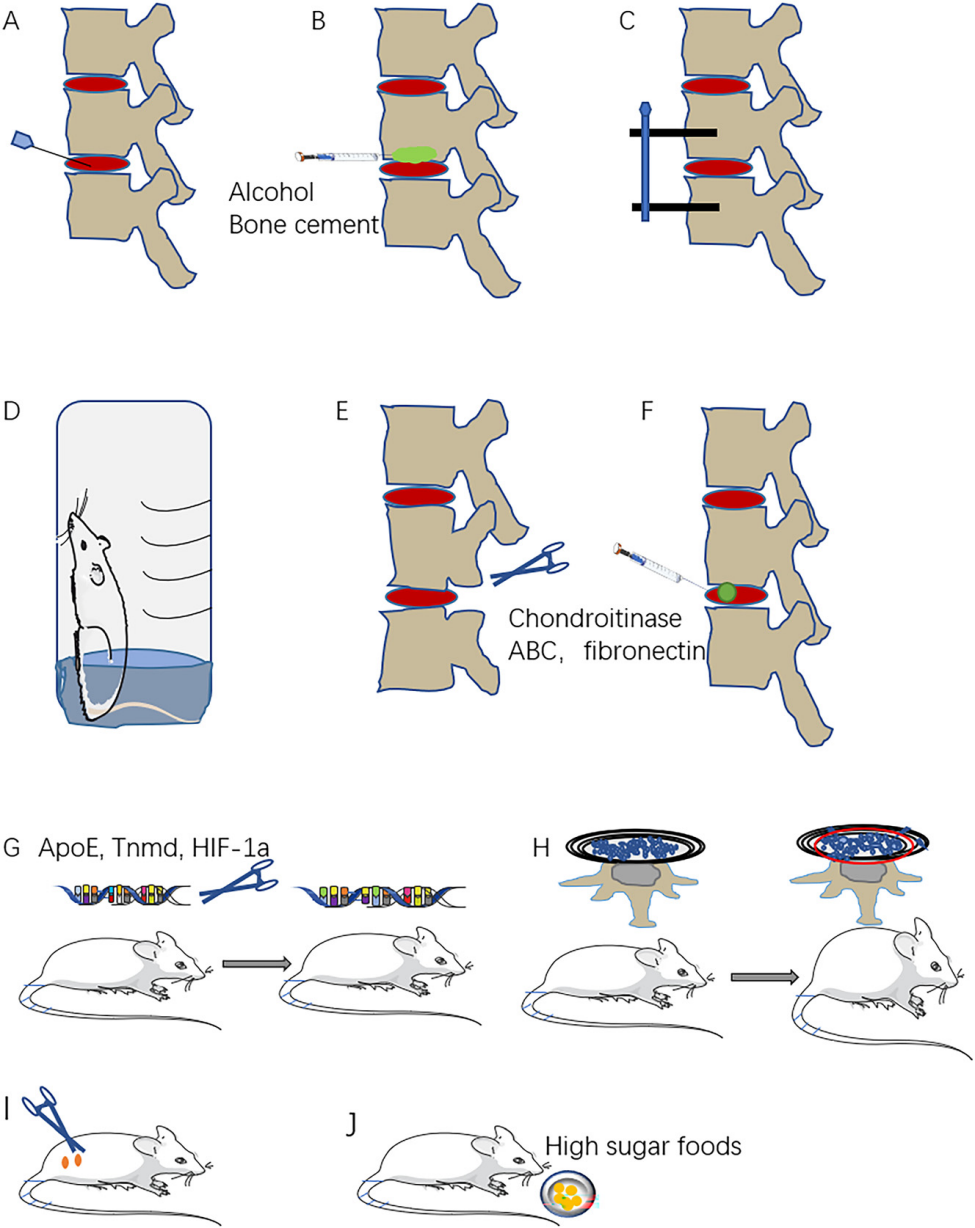
Examples of animal models for intervertebral disc degeneration. (A) Annulus fibrosus and nucleus pulposus injury model. (B) Cartilage endplate injury model. (C) Compression model. (D) Upright model. (E) Spinal instability model. (F) Chemically induced model. (G) Gene knockout model. (H) Spontaneous degeneration model. (I) ovariectomy model. (J) High sugar diet induced type 2 diabetes triggered intervertebral disc degeneration model.

The method of creating animal models through NP and AF puncture is relatively simple, highly reproducible, and successfully mimics the human processes of NP injury, AF injury, and subsequently IDD. This makes it suitable for investigating the pathophysiological mechanisms following AF injury and acute intervertebral disc herniation (Figure 2A). However, this method has limitations, including the need for long observation periods, the potential for infection after needle insertion, and the inherent possibility that the puncture itself may trigger an immune inflammatory response.

#### 3.1.2 Cartilage endplate injury

The intervertebral disc is one of the largest non-vascular and nerve organs in the vertebrate body, with nutrients permeating from the CEP and outer AF to the NP tissue. As the IDD, the supply of nutrients decreases, limiting intervertebral disc cell activity and viability [26]. Therefore, a model of disc degeneration constructed by blocking the CEP blood supply to reduce the nutrient supply to the disc can be used to explore the relationship between nutrient supply and IDD (Figure 1).

Injection of anhydrous alcohol beneath the caudal CEP of rat discs induced a transformation of the NPCs, shifting their morphology from cord-like to cartilage-like, and finally into fibrocartilage cells. This process was associated with rupture of the annulus fibrosus, followed by degradation and eventual disappearance of the CEP growth plate [27]. Another study utilizing an immature animal model investigated the effects of blood supply blockade using bone cement on IDD (Figure 2B). They found that injecting bone cement into the CEP effectively induced IDD [28].

Researchers have established IDD models by administering the chemical drugs pingyangmycin into the subchondral bone of the lumbar disc in rhesus monkeys and rabbits under CT guidance, thereby blocking nutrient supply. Postoperative observations revealed the formation of bony endplates, a reduction in intervertebral disc height, and alterations in MRI signals [29], 30]. These findings suggest that CEP is essential for maintaining the viability of intervertebral disc cells.

### 3.2 Abnormal mechanical stimulation model

Biomechanics is fundamental to intervertebral disc homeostasis, and abnormal mechanical loading is a primary driver of disc degeneration. For instance, individuals engaged in heavy labor face a significantly elevated risk of disc degeneration. Disrupting the spine’s biomechanical equilibrium can alter the morphological and physiological characteristics of the disc, mirroring the early stages of disc degeneration observed in humans.

#### 3.2.1 Compression model

Fixation of lumbar vertebrae through pedicle screws and rods leads to changes in the intervertebral disc load environment, thereby inducing IDD [31] (Figure 2C). Examination of the 1.8 N and 4.5 N load groups identified structural disorganization within both the NP and AF, occurring on both convex and concave surfaces. MRI and histological findings demonstrated that the degree of IDD was amplified by greater compression forces. Moreover, the compression model exhibited increased mRNA expression of MMP-3 and MMP-13, with a concurrent significant decrease in collagen type II-α1 expression [32]. Piezo1 expression was upregulated by excessive compression loading, resulting in ECM degradation and increased NP cell apoptosis. Inhibition of Piezo1 attenuated these pathological processes [33].

By circumferentially coiling and fixing rat tails with steel wire, Sakai et al. were able to subject each intervertebral disc to varying degrees of compressive stress. This resulted in the development of IDD models with different levels of degeneration [34]. The study by Zak et al. demonstrated that long-term cyclic compression-flexion loading (100,000 cycles) leads to degenerative changes in both the structural and mechanical properties of intervertebral discs, while highlighting the posterior column’s crucial role in preserving the integrity and biomechanical characteristics of the annulus fibrosus [35].

#### 3.2.2 Bipedal animal model

The upright walking posture of humans is thought to be an important mechanical factor contributing to disc degeneration (Figure 2D). In mice, the movement pattern during standing and jumping is similar to that of humans. In addition, some researchers induce IDD by forcing animals to stand on both feet to mimic human bipedal gait after amputation of their forelimbs [36]. As the time and biomechanical load increases, the fissure at the AF gradually increases, the height of both the disc and CEP decreases, and the extracellular matrix composition changes, forming an IDD model [37]. Researchers leveraged mice’s innate fear of water to induce them to adopt a bipedal stance within a water-filled enclosure. The water temperature was maintained at 24 °C with a depth of 5 mm to ensure the animals’ fur remained dry and their body temperature stayed normal. After 10 weeks, the mice exhibited severe IDD, primarily affecting the AF and facet joints. This method established a non-invasive and effective IDD model that closely mimics the human condition. However, it necessitates continuous monitoring of water temperature and volume, and the model development period is relatively long [38].

#### 3.2.3 Spinal instability models

This model induced spinal instability by disrupting the dynamic and static equilibrium of the spine and damage to peri-vertebral disc structures, causing excessive spinal motion (Figure 2E). The resulting biomechanical imbalance, caused by removing the paravertebral musculature and posterior ligaments, led to spinal instability. This instability subsequently drove CEP hyperplasia, increased NP apoptosis, and a reduction in disc height, successfully establishing an IDD model [39]. After 12 weeks following facetectomy (mechanical instability), intervertebral disc degeneration occurred, with a decrease in type II collagen expression, while the model promoted the collapse of subchondral bone trabeculae [40]. Liu et al. reported a mouse model of lumbar instability, in which L3–L5 spinous processes were surgically exposed, paraspinal muscles were separated, articular processes and ligaments were removed, and degenerative changes began to appear in the L4/5 intervertebral discs one week after surgery [41]. Oichi et al. created a lumbar instability model by surgically excising the lumbar facet joints, supraspinous ligament, and interspinous ligaments of mice. Imaging studies showed a decrease in disc height beginning 2 weeks after surgery, with continued decline over the next 12 weeks. Furthermore, histological analysis confirmed the presence of IDD in the lumbar vertebrae [42].

However, the modelling time for this model is relatively long, the cost of the experiment is high, and it remains controversial whether its degenerative factors include surgical stimulation. Unlike models that directly injure the disc, these animal models preserve the integrity of the annulus fibrosus and nucleus pulposus. The slow progression of IDD makes these models more representative of adjacent segment degeneration seen after human lumbar fusion surgery [43].

### 3.3 Chemically induced model

Biochemical reagents have been used to induce animal models for disc degeneration. Intralesional injection of monosodium iodoacetate into the NP of the rat intervertebral disc revealed a narrowing of the intervertebral space and a decrease in the signal intensity of magnetic resonance T2-weighted images after 6 weeks, suggesting degeneration of the intervertebral disc [44]. Rustenburg et al. injected collagenase and chondroitinase ABC into the intervertebral disc of caprine, causing degeneration and necrosis of the NP, AF, CEP. It was found that extracellular matrix components decreased, while degradative enzymes and catabolic proteins (IL-1β, IL-8, and VEGF) continued to increase [15]. Another method was to inject 100 μg of trypsin into the intervertebral disc of bovine to lyse the proteoglycan and collagen (Figure 2F). Once in the disc centre, the trypsin solution was slowly injected and the needle gradually pulled out to avoid flow [45]. Liu et al. used a minimally invasive injection technique to inject fibronectin fragment into the intervertebral disc of rabbits. IDD began to occur 4 weeks after surgery, with increased expression of MMPs, iNOS and TNF-1 (Figure 1). With prolonged postoperative observation, the NP was completely replaced by fibrous tissue, successfully establishing a reproducible, simple and cost-effective model of disc degeneration [46].

Although chemical induced models are less invasive, reproducible, and allow for easy drug administration, their limitations stem from the primarily physiological nature of IDD. The natural progression of degeneration is not effectively mimicked by the injection of chemical reagents. Moreover, the distribution of the injected drugs throughout the intervertebral disc and their impact on subsequent experimental assessments must be carefully evaluated.

### 3.4 Gene knockout model

In recent years, with the development of genetic engineering technology and the progress of gene knockout technology, it provides a new method for animal models of many diseases. In particular, crispr-cas9 gene editing technology is becoming more and more mature, and it is possible to make a large number of degenerative disease models [47], [48], [49], [50]. After ApoE gene knockout in rabbits, it was found that the inflammatory factors in the intervertebral disc were increasing and the metabolism in the intervertebral disc was disordered, resulting in the symptoms of IDD [51]. Knockout of the mice tenomodulin (tnmd) gene promoted the upregulation of p65 and matrix metalloproteinases, induced angiogenesis and macrophage infiltration in the outer AF, and increased chondroid NPCs in the NP [52]. Some scholars have studied the role of HIF-1α gene in IDD in mice (Figure 2G). The results show that glycosaminoglycan, type II collagen and the expression of vascular endothelial growth factor are reduced in the gene knockout HIF-1α group [53]. The secreted protein, acidic, rich in cysteine (SPARC)-null mice exhibited decreased tolerance to axial stretching, hindpaw cold hypersensitivity, motor impairment, and the degree of IDD was more severe compared with age-matched control mice [54].

### 3.5 Spontaneous degeneration model

Some animals show disc changes with age, which have many common characteristics with human disc degeneration (Figure 2H). Sand rat are a classic model of spontaneous disc degeneration. It is found that the disc degeneration of 2-month-old sand rat is similar to that of humans [55]. Vincent et al. also found that 24-month-old mice exhibited typical disc degeneration compared to 3- and 12-month-old mice [56]. Choi et al. found in SM/J mice a progressive decrease in NPCs, changes in stromal composition and a decrease in disc height with age, similar to many of the distinguishing features of human disc degeneration, a new small animal model of disc degeneration [57].

Our early experiments found that spontaneous degeneration of intervertebral disc also existed in rats. Compared with the 4W groups, the level of vacuolated nochordal cells (50 weeks) increased gradually over time, and nucleus pulposus cell clusters and serpentine AF were found, intervertebral disc height decreased significantly, Pfirrmann grade increased, indicating disc generation [58].

### 3.6 Other IDD models

Exposure to tobacco smoke reduces proteoglycan content in the intervertebral discs of mice and significantly enhances matrix metalloproteinase activity. This smoke exposure also resulted in a two-fold increase in senescent cells within the intervertebral discs of the mice [59]. Removal of ovaries in rats reduce disc height, water content, and histologic score [60] (Figure 2I). In addition, in rats with type 2 diabetes, metabolic disorders in intervertebral disc can lead to disc degeneration [61] (Figure 2J).

## 4 Conclusions and the future

IDD is one of the main causes of chronic low back pain and neurological dysfunction. Its pathological mechanisms are complex, involving inflammation, oxidative stress, extracellular matrix degradation, and cellular aging. Animal models serve as important tools for studying IDD, playing a crucial role in revealing disease mechanisms and evaluating potential therapies. Regarding the selection of experimental animals, from the perspective of human lifestyle and spinal morphology, the spinal physiological structure and biomechanical environment of large animals (such as goats, pigs, primates) share a high degree of similarity with humans [62], [63], [64]. Factors like spontaneous degeneration and spinal mechanical stress also closely resemble human conditions. However, due to considerations such as cost, maintenance requirements, and ethical constraints, large primates are difficult to become routine models for intervertebral disc degeneration. Currently, rodents and rabbits, benefiting from their low husbandry costs and readily available supply, remain the preferred experimental animals for intervertebral disc degeneration models. Existing animal models of IDD each have their unique advantages and limitations, making it difficult to fully replicate the natural course and heterogeneity of human disc degeneration (Table 1). Various intervertebral disc degeneration models still lack standardized protocols (e.g., puncture depth, enzyme dosage, gene knockout strategies, etc.), which leads to discrepancies in results and makes inter-study comparisons difficult. Researchers need to develop detailed standardized operating procedures and unified reporting guidelines specifically for different animal models (e.g., rabbits, mice, etc.).

**Table 1:** Comparative evaluation table for animal models of intervertebral disc degeneration.

Induction method	Concrete method	Study	Speciman	Observed onset of degeneration	Advantages	Limitations	Suitable research fields
Intervertebral disc injury model	Needle puncture	Glaeser [19]	SD rat	4 and 8 weeks	1. Relatively simple and reproducible 2. Suitable for disc injury disease	1. Long observation period 2. Infection 3. Immune inflammatory response	Regenerative therapy
	Needle puncture	Gianluca [20]	sheep	1, 3, and 6 months			Regenerative therapy
	Needle puncture	Zhang [21]	SD rat	4 weeks			Regenerative therapy/pharmacological testing
	Needle puncture	Zhang [22]	SD rat	4 weeks			Regenerative therapy
	Needle puncture and ethanol injection	Yuan [27]	SD rat	1, 2, 3, and 6 months			Regenerative therapy
	Cement in CEP	Kang [28]	Pig	3 months			Regenerative therapy/pharmacological testing
	Pingyangmycin in CEP	Wei [29]	Monkey	1, 3, 6, 9, 12, and 15 months			Regenerative therapy/pharmacological testing
	Pingyangmycin in CEP	Wei [30]	Rabbit	1, 3, and 6 months			Regenerative therapy/pharmacological testing
Abnormal mechanical stimulation model	Immobilization	Wang [31]	Sheep	6 and 26 weeks	1. Without acute injury 2. Closely representing the scenario common in human	1. Surgical procedure exacerbates spinal instability 2. Static bending and compression	Regenerative therapy/pharmacological testing
	Compression load	Ji [32]	SD rat	14 days			Regenerative therapy/pharmacological testing
	Compression load	Li [33]	Mice	2 months			Regenerative therapy
	Tail-looping	Sakai [34]	Mice	4, 8, and 12 weeks			Regenerative therapy
Bipedal animal model	Forelimbs amputation	Kong [36]	SD rat	6 months	Simulate the upright state of the human	1. Muscle tissue damage worsens 2. Animal ethics controversial	Regenerative therapy
	Limited water-containing space	Ao [38]	Mice	6 and 10 weeks			Regenerative therapy/pharmacological testing
	Limited water-containing space	Jin [37]	Mice	10 weeks			Regenerative therapy
Spinal instability models	Paraspinal musculature was exposed and excised	Yao [39]	Mice	4 and 8 weeks	1. Creating static and dynamic posterior instability 2. Mimic the mechanics engaged in the onset and natural course of IDD 3. Strong operability 4. Relatively short period of IDD development	1. Modelling time relatively long 2. The cost of experimental equipment is relatively high 3. Muscle tissue damage	Regenerative therapy/pharmacological testing
	Lumbar facetectomy	Xiao [40]	Mice	12 weeks			Regenerative therapy/pharmacological testing
	Cutting off interspinous ligaments	Liu [41]	Mice	1, 2 and 16 weeks			Regenerative therapy/pharmacological testing
	Resection of facet joints, supra- and interspinous ligaments	Oichi et al. [42]	Mice	2 and 12 weeks			Regenerative therapy
Chemically induced model	Monosodium iodoacetate	Najah [44]	SD rat	6 weeks	1. Less invasive, 2. Reproducible 3. Easy to administer drug doses	1. Chemical reagents cannot replicate the natural progression of IDD 2. Distribution is generally uneven	Regenerative therapy/pharmacological testing
	Chondroitinase ABC	Rustenburg [15]	Caprine	1week			Regenerative therapy/pharmacological testing
	Trypsin	Rosenzweig [45]	Bovine	1week			
	Fibronectin fragment	Liu [46]	Rabbit	4, 8, 12, and 16 weeks			Regenerative therapy
Spontaneous degeneration model	Spontaneous degeneration	Gruber [55]	Sand rat	2 months	Closer to human disc physiopathology	Long time consumption	Regenerative therapy
	Spontaneous degeneration	Vincent [56]	Mice	24 months			Regenerative therapy
	Spontaneous degeneration	Choi [57]	Mice	8 and 17 weeks			Regenerative therapy
		Zhang [58]	SD rat	50 weeks			Regenerative therapy
Gene knockout models	APOE knockout	Beierfuβ [51]	Rabbit	24 months	1. Revealing the role of specific genes in IDD 2. Clear genetic background 3. Relatively uniform genetic background	1. Knockout of certain genes may lead to embryonic death or defects 2. Long time consumption	Regenerative therapy/pharmacological testing
	Tnmd knockout	Lin [52]	Mice	6 months			Regenerative therapy/pharmacological testing
	HIF-1 alpha knockout	Wu [53]	Mice	6 and 12 weeks			Regenerative therapy/pharmacological testing
	SPARC knockout	Magali [54]	Mice	78 weeks			Regenerative therapy/pharmacological testing
Others	Smoking	Wang [59]	Mice	6 months	Further support tobacco smoke as a contributor to spinal degeneration	1. The molding time is long. 2. Smoke induced osteoporosis	Regenerative therapy
	Ovariectomy	Najah [60]	SD rat	3 and 6 weeks	1. Ease of establishment 2. Relatively low cost 3. Mimics hormonal changes	1. Non-physiological 2. Sex-specific 3. Animal welfare concerns	Pharmacological testing
	Type 2 diabetes	Rosenberg [61]	SD rat	68 days	Further validates the causality between type 2 diabetes and IDD	1. Not a full mimic of human IDD 2. Long time consumption	Regenerative therapy/pharmacological testing

To evaluate the effectiveness of novel regenerative strategies for the NP, preclinical models are needed that preserve the AF of the intervertebral disc. With this in mind, the study was to characterize a preclinical ovine model of IDD. This model was induced by damaging and repairing the EP via a transpedicular approach [20]. In cases of mild disc degeneration, defined by biochemical imbalances and microenvironmental disruption without structural collapse, biomaterial-based strategies aim to restore disc homeostasis. Hydrogels, such as methacrylated cellulose derivatives or alginate-pNIPAAm composites, are employed for injection due to their ability to mimic the nucleus pulposus (NP) and annulus fibrosus (AF). Their mechanism of action involves regulating hypoxia, acidity, and inflammation by localized delivery of anti-inflammatory agents or growth factors. This delivery suppresses catabolic enzymes like MMP-3 and ADAMTS-4, while simultaneously encouraging the synthesis of extracellular matrix components, including aggrecan and collagen II [65], [66], [67]. Many treatment strategies, such as stem cell therapy and biomaterial repair, have shown significant effects in animal experiments but have failed to meet expectations in clinical trials [67]. With the advancement of biomedical technology and the deepening understanding of the IDD mechanism, some new models have been proven to have good advantages in regenerative research. At the same time, new models will be created in the future, such as organoid models of nucleus pulposus [68], microfluidic chemotaxis intervertebral disc organ-on-a-chip simulating intervertebral disc microenvironments [69], humanized animal models or patient-derived xenografts for personalized medicine.

Therefore, ongoing refinement of IDD animal models will promote a deeper understanding of the disease mechanisms and lay the foundation for the development of novel therapeutic approaches.

## References

[1] Melrose J, Guilak F (2024). Diverse and multifunctional roles for perlecan (HSPG2) in repair of the intervertebral disc. JOR Spine.

[2] Ambrosio L, Schol J, Ruiz-Fernández C, Tamagawa S, Joyce K, Nomura A (2024). Getting to the core: exploring the embryonic development from notochord to nucleus pulposus. J Dev Biol.

[3] Yang S, Zhang F, Ma J, Ding W (2020). Intervertebral disc ageing and degeneration: the antiapoptotic effect of oestrogen. Ageing Res Rev.

[4] Wang Y, Kang J, Guo X, Zhu D, Liu M, Yang L (2022). Intervertebral disc degeneration models for pathophysiology and regenerative therapy-benefits and limitations. J Invest Surg.

[5] Tian T, Wang H, Li Z, Yang S, Ding W (2021). Intervertebral disc degeneration induced by needle puncture and ovariectomy: a rat coccygeal model. BioMed Res Int.

[6] Wang D, Lai A, Gansau J, Seifert AC, Munitz J, Zaheer K (2023). Lumbar endplate microfracture injury induces Modic-like changes, intervertebral disc degeneration and spinal cord sensitization - an in vivo rat model. Spine J.

[7] Wang J, Fan C, Zhang Y, Hua D, Ji Z, He W (2025). (Z)-ligustilide alleviates intervertebral disc degeneration by suppressing nucleus pulposus cell pyroptosis via Atg5/NLRP3 axis. Sci China Life Sci.

[8] Herrera Quijano MA, Sharma N, Morissette Martin P, Séguin CA, Flynn LE (2022). Development of 2-D and 3-D culture platforms derived from decellularized nucleus pulposus. Front Bioeng Biotechnol.

[9] Ouyang A, Cerchiari AE, Tang X, Liebenberg E, Alliston T, Gartner ZJ (2017). Effects of cell type and configuration on anabolic and catabolic activity in 3D co-culture of mesenchymal stem cells and nucleus pulposus cells. J Orthop Res.

[10] Zhang Y, Tan W, Wu M, Sun J, Cao W, Zhou CS (2021). Characterization and cytocompatibility of 3D porous biomimetic scaffold derived from rabbit nucleus pulposus tissue in vitro. J Mater Sci Mater Med.

[11] Dai X, Guan Y, Zhang Z, Xiong Y, Liu C, Li H (2021). Comparison of the differentiation abilities of bone marrow-derived mesenchymal stem cells and adipose-derived mesenchymal stem cells toward nucleus pulposus-like cells in three-dimensional culture. Exp Ther Med.

[12] McDonnell EE, Buckley CT (2022). Two- and three-dimensional in vitro nucleus pulposus cultures: an in silico analysis of local nutrient microenvironments. JOR Spine.

[13] Paillat L, Coutant K, Dutilleul M, Le Lay S, Camus A (2023). Three-dimensional culture model to study the biology of vacuolated notochordal cells from mouse nucleus pulposus explants. Eur Cell Mater.

[14] Gawri R, Mwale F, Ouellet J, Roughley PJ, Steffen T, Antoniou J (2011). Development of an organ culture system for long-term survival of the intact human intervertebral disc. Spine.

[15] Rustenburg CM, Snuggs JW, Emanuel KS, Thorpe A, Sammon C, Le Maitre CL (2020). Modelling the catabolic environment of the moderately degenerated disc with a caprine *ex vivo* loaded disc culture system. Eur Cell Mater.

[16] Stannard JT, Edamura K, Stoker AM, O’Connell GD, Kuroki K, Hung CT (2016). Development of a whole organ culture model for intervertebral disc disease. J Orthop Transl.

[17] Gallate ZS, D’Erminio DN, Nasser P, Laudier DM, Iatridis JC (2023). Galectin-3 and RAGE differentially control advanced glycation endproduct-induced collagen damage in murine intervertebral disc organ culture. JOR Spine.

[18] Kanan M, Eby O, Kelkar A, Serhan H, Zodak Y, Aldoohan S (2022). Electrical stimulation-mediated tissue healing in porcine intervertebral disc under mechanically dynamic organ culture conditions. Spine.

[19] Glaeser JD, Tawackoli W, Ju DG, Yang JH, Kanim LE, Salehi K (2020). Optimization of a rat lumbar IVD degeneration model for low back pain. JOR Spine.

[20] Vadalà G, Russo F, De Strobel F, Bernardini M, De Benedictis GM, Cattani C (2018). Novel stepwise model of intervertebral disc degeneration with intact annulus fibrosus to test regeneration strategies. J Orthop Res.

[21] Zhang C, Wang F, Xie Z, Chen L, Sinkemani A, Yu H (2018). Dysregulation of YAP by the Hippo pathway is involved in intervertebral disc degeneration, cell contact inhibition, and cell senescence. Oncotarget.

[22] Zhang C, Wang F, Gao Z, Zhang P, Gao J, Wu X (2020). Regulation of Hippo signaling by mechanical signals and the cytoskeleton. DNA Cell Biol.

[23] Lv X, Chen S, Gao F, Hu B, Wang Y, Ni S (2021). Resveratrol-enhanced SIRT1-mediated osteogenesis in porous endplates attenuates low back pain and anxiety behaviors. Faseb J.

[24] Yuan HJ, Wang CY, Wang YF (2021). Endoscopic joint capsule and articular process excision to treat lumbar facet joint syndrome: a case report. World J Clin Cases.

[25] Lee S, Millecamps M, Foster DZ, Stone LS (2020). Long-term histological analysis of innervation and macrophage infiltration in a mouse model of intervertebral disc injury-induced low back pain. J Orthop Res.

[26] Chen L, Xie ZY, Liu L, Zhu L, Wang F, Fan P (2019). Nuclear factor-kappa B-dependent X-box binding protein 1 signalling promotes the proliferation of nucleus pulposus cells under tumour necrosis factor alpha stimulation. Cell Prolif.

[27] Yuan W, Che W, Jiang YQ, Yuan FL, Wang HR, Zheng GL (2015). Establishment of intervertebral disc degeneration model induced by ischemic sub-endplate in rat tail. Spine J.

[28] Kang R, Li H, Ringgaard S, Rickers K, Sun H, Chen M (2014). Interference in the endplate nutritional pathway causes intervertebral disc degeneration in an immature porcine model. Int Orthop.

[29] Wei F, Zhong R, Wang L, Zhou Z, Pan X, Cui S (2015). Pingyangmycin-induced in vivo lumbar disc degeneration model of rhesus monkeys. Spine.

[30] Wei F, Zhong R, Pan X, Khaleel M, Hammoud A, Zhou Z (2019). Computed tomography-guided sub-end plate injection of pingyangmycin for a novel rabbit model of slowly progressive disc degeneration. Spine J.

[31] Wang T, Pelletier MH, Christou C, Oliver R, Mobbs RJ, Walsh WR (2018). A novel *in vivo* large animal model of lumbar spinal joint degeneration. Spine J.

[32] Ji Y, Zhu P, Zhang L, Yang H (2021). A novel rat tail disc degeneration model induced by static bending and compression. Animal Model Exp Med.

[33] Li F, Chen M, Zhang M, Chen S, Qu M, He S (2025). Targeting Piezo1 channel to alleviate intervertebral disc degeneration. J Orthop Transl.

[34] Sakai D, Nishimura K, Tanaka M, Nakajima D, Grad S, Alini M (2015). Migration of bone marrow-derived cells for endogenous repair in a new tail-looping disc degeneration model in the mouse: a pilot study. Spine J.

[35] Żak M, Pezowicz C (2021). Effect of overload on changes in mechanical and structural properties of the annulus fibrosus of the intervertebral disc. Biomech Model Mechanobiol.

[36] Kong M, Zhang Y, Song M, Cong W, Gao C, Zhang J (2021). Myocardin-related transcription factor A nuclear translocation contributes to mechanical overload-induced nucleus pulposus fibrosis in rats with intervertebral disc degeneration. Int J Mol Med.

[37] Jin LY, Yin HL, Xu YQ, Xu S, Song XX, Luo Y (2023). Long-term whole-body vibration induces degeneration of intervertebral disc and facet joint in a bipedal mouse model. Front Bioeng Biotechnol.

[38] Ao X, Wang L, Shao Y, Chen X, Zhang J, Chu J (2019). Development and characterization of a novel bipedal standing mouse model of intervertebral disc and facet joint degeneration. Clin Orthop Relat Res.

[39] Yao S, Li Y, Ruan H, Wu L, Zeng H (2025). Gubi decoction ameliorates porous cartilage endplate in an intervertebral disc degeneration model mouse through inhibition of NF-κB activity and pyroptosis. J Inflamm Res.

[40] Xiao ZF, Su GY, Hou Y, Chen SD, Zhao BD, He JB (2020). Mechanics and biology interact in intervertebral disc degeneration: a novel composite mouse model. Calcif Tissue Int.

[41] Liu S, Sun Y, Dong J, Bian Q (2021). A mouse model of lumbar spine instability. J Vis Exp.

[42] Oichi T, Taniguchi Y, Soma K, Chang SH, Yano F, Tanaka S (2018). A mouse intervertebral disc degeneration model by surgically induced instability. Spine.

[43] Fukui D, Kawakami M, Cheng K, Murata K, Yamada K, Sato R (2017). Three-dimensional micro-computed tomography analysis for spinal instability after lumbar facetectomy in the rat. Eur Spine J.

[44] Elmounedi N, Bahloul W, Guidara AR, Aoui M, Trigui M, Keskes H (2023). Establishment of an animal model of disk degeneration by intradiskal injection of monosodium iodoacetate. World Neurosurg.

[45] Rosenzweig DH, Fairag R, Mathieu AP, Li L, Eglin D, D’Este M (2018). Thermoreversible hyaluronan-hydrogel and autologous nucleus pulposus cell delivery regenerates human intervertebral discs in an ex vivo, physiological organ culture model. Eur Cell Mater.

[46] Liu HF, Zhang H, Qiao GX, Ning B, Hu YL, Wang DC (2013). A novel rabbit disc degeneration model induced by fibronectin fragment. Jt Bone Spine.

[47] Xi Z, Vats A, Sahel JA, Chen Y, Byrne LC (2022). Gene augmentation prevents retinal degeneration in a CRISPR/Cas9-based mouse model of PRPF31 retinitis pigmentosa. Nat Commun.

[48] Lu ZY, Chen PB, Xu QY, Li B, Jiang SD, Jiang LS (2023). Constitutive and conditional gene knockout mice for the study of intervertebral disc degeneration: current status, decision considerations, and future possibilities. JOR Spine.

[49] Voisard P, Diofano F, Glazier AA, Rottbauer W, Just S (2022). CRISPR/Cas9-Mediated constitutive loss of VCP (valosin-containing protein) impairs proteostasis and leads to defective striated muscle structure and function in vivo. Int J Mol Sci.

[50] Liu S, Zhou M, Ruan Z, Wang Y, Chang C, Sasaki M (2021). AIF3 splicing switch triggers neurodegeneration. Mol Neurodegener.

[51] Beierfuß A, Hunjadi M, Ritsch A, Kremser C, Thomé C, Mern DS (2019). APOE-knockout in rabbits causes loss of cells in nucleus pulposus and enhances the levels of inflammatory catabolic cytokines damaging the intervertebral disc matrix. PLoS One.

[52] Lin D, Alberton P, Delgado Caceres M, Prein C, Clausen-Schaumann H, Dong J (2020). Loss of tenomodulin expression is a risk factor for age-related intervertebral disc degeneration. Aging Cell.

[53] Wu WJ, Zhang XK, Zheng XF, Yang YH, Jiang SD, Jiang LS (2013). SHH-dependent knockout of HIF-1 alpha accelerates the degenerative process in mouse intervertebral disc. Int J Immunopathol Pharmacol.

[54] Millecamps M, Czerminski JT, Mathieu AP, Stone LS (2015). Behavioral signs of axial low back pain and motor impairment correlate with the severity of intervertebral disc degeneration in a mouse model. Spine J.

[55] Gruber HE, Johnson TL, Leslie K, Ingram JA, Martin D, Hoelscher G (2002). Autologous intervertebral disc cell implantation: a model using Psammomys obesus, the sand rat. Spine.

[56] Vincent K, Mohanty S, Pinelli R, Bonavita R, Pricop P, Albert TJ (2019). Aging of mouse intervertebral disc and association with back pain. Bone.

[57] Choi H, Tessier S, Silagi ES, Kyada R, Yousefi F, Pleshko N (2018). A novel mouse model of intervertebral disc degeneration shows altered cell fate and matrix homeostasis. Matrix Biol.

[58] Zhang C, Wang F, Xie Z, Chen L, Wu X (2021). The hippo pathway orchestrates cytoskeletal organisation during intervertebral disc degeneration. Acta Histochem.

[59] Wang D, Nasto LA, Roughley P, Leme AS, Houghton AM, Usas A (2012). Spine degeneration in a murine model of chronic human tobacco smokers. Osteoarthr Cartil.

[60] Elmounedi N, Bahloul W, Aoui M, Sahnoun N, Ellouz Z, Keskes H (2023). Original animal model of lumbar disc degeneration. Libyan J Med.

[61] Rosenberg JL, Schaible E, Bostrom A, Lazar AA, Graham JL, Stanhope KL (2023). Type 2 diabetes impairs annulus fibrosus fiber deformation and rotation under disc compression in the University of California Davis type 2 diabetes mellitus (UCD-T2DM) rat model. PNAS Nexus.

[62] Rajagopal K, Schaer TP, Meadows KD, Boyes M, Hilliard R, O’Donnell JC (2025). *In vivo* measurements reveal increased nucleus pulposus lactate and oxygen concentrations in a goat model of intervertebral disc degeneration. JOR Spine.

[63] Asl MMS, Goodarzi N, Soroori S (2025). Morphometric and morphological study of thoracic and lumbar intervertebral discs in guinea pigs (Cavia porcellus). Anat Histol Embryol.

[64] Zhou L, Li H, Chen C, Yang H, Zhang G, Zhang Q (2025). A quality by design (QbD) project of human dermal fibroblast and its therapeutic effects on managing degenerative intervertebral disc fibrosis in rabbit and cynomolgus monkey. Front Biosci.

[65] Vasiliadis ES, Pneumaticos SG, Evangelopoulos DS, Papavassiliou AG (2014). Biologic treatment of mild and moderate intervertebral disc degeneration. Mol Med.

[66] Liu Y, Zhao Z, Guo C, Huang Z, Zhang W, Ma F (2023). Application and development of hydrogel biomaterials for the treatment of intervertebral disc degeneration: a literature review. Front Cell Dev Biol.

[67] Ogaili RH, Alassal A, Za’aba NF, Zulkiflee I, Mohd Isa IL (2025). Regenerative strategies for intervertebral disc degeneration. J Orthop Transl.

[68] Zhang C, Jing Y, Wang J, Xia Z, Lai Y, Bai L (2024). Skeletal organoids. Biomater Transl.

[69] Son HG, Hwang MH, Lee S, Kim AG, Kim TW, Kim JH (2023). Intervertebral disc organ-on-a-chip: an innovative model to study monocyte extravasation during nucleus pulposus degeneration. Lab Chip.

